# Clinical application of 3D topographic device for monitoring scoliosis progression

**DOI:** 10.1186/1748-7161-8-S2-O23

**Published:** 2013-09-18

**Authors:** John Thometz, XueCheng Liu, Channing Tassone, Roger Lyon

**Affiliations:** 1 Dept. of Orthopaedic Surgery, Children's Hospital of WI. Medical College of WI, Milwaukee, WI USA

## Background

Cumulative exposure to radiation from diagnostic radiographs increases patient risk of cancer development. Minimizing exposure to radiation is desired for the patient’s health, so there is a need to develop alternative non-invasive tools to measure spine deformity.

## Purpose

The aims of this study were to (1) determine the reproducibility of the newly developed 3-dimensional (3D) Milwaukee Topographic System (MTS) through inter- and intra-rater measurements and (2) calculate the correlation between the 3D angle obtained by the device and the Cobb angle measured with radiographs.

## Methods

The study group consisted of twenty children with idiopathic scoliosis (IS), aged 6-18 years, with a range of Cobb angles. The MTS is composed of two wide-angle optical cameras, two electro-magnetic sensors, a light, a software package, a positioning frame, and a desktop computer. The device required four 5-second scan sweeps (three vertical and one horizontal) for each subject. Four measurements were performed by two investigators, alternately. Reliability for the device was measured with intra-class correlation coefficient (ICC) controlling subject effect in a stratified model. Pearson correlations were calculated as well as mean values and confidence intervals for each metric.

## Results

A Pearson data analysis showed excellent intra-class correlation (ICC > 0.6) between investigators for 10 metrics, and moderate ICC (from 0.4 to 0.6) for four metrics (p<0.05). A Pearson analysis of intra-investigator ICC demonstrated moderate to excellent ICC in all 17 measured parameters (p<0.05). A Pearson correlation coefficient between the 3D angles obtained from the MTS and radiographs was remarkably higher for the Cobb angle in the sagittal plane (r=0.91, p<0.001).

## Conclusions and discussion

The new MTS provides reproducible measures for the assessment of patients with scoliotic deformity.

